# Supplementary material for: The interactome of intact mitochondria by cross-linking mass spectrometry provides evidence for coexisting respiratory supercomplexes
Source: Mol Cell Proteomics. 2017 Dec 8;17(2):216–32. doi: 10.1074/mcp.RA117.000470 (PMC5795388; doi:10.1074/mcp.RA117.000470)
Supplement: Supplemental Data [file supp_RA117.000470_133922_0_supp_23978_fzffwf.zip › spectra_annotation/mito_spectra_annotation/366-1-4-1-28-1.pdf]

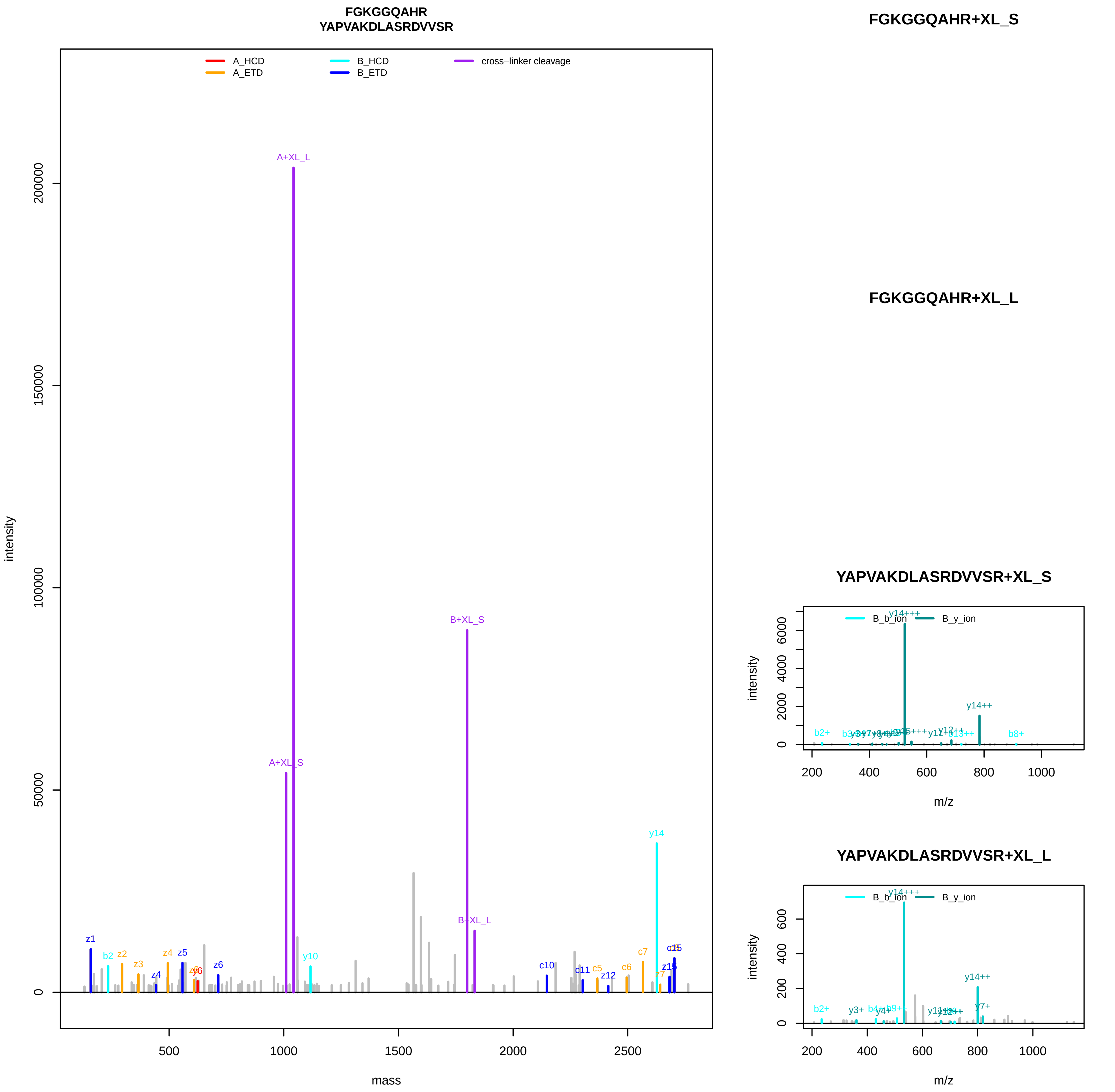
FGKGGQAHR
YAPVAKDLASRDVVSR
A_HCD
A_ETD
B_HCD
B_ETD
cross−linker cleavage
intensity
mass
0
50000
100000
150000
200000
500
1000
1500
2000
2500
A+XL_L
A+XL_S
B+XL_S
B+XL_L
z1
b2
z2
z3
z4
z5
y6
z6
y10
c10
c11
c5
z12
c6
c7
z7
y14
z15
c15
FGKGGQAHR+XL_S
FGKGGQAHR+XL_L
YAPVAKDLASRDVVSR+XL_S
YAPVAKDLASRDVVSR+XL_L
B_b_ion
B_y_ion
m/z
y14+++
y14++
b2+
b8+
y7+
200
400
600
800
1000
